# Low-level EMF effects on wildlife and plants: What research tells us about an ecosystem approach

**DOI:** 10.3389/fpubh.2022.1000840

**Published:** 2022-11-25

**Authors:** B. Blake Levitt, Henry C. Lai, Albert M. Manville

**Affiliations:** ^1^National Association of Science Writers, Berkeley, CA, United States; ^2^Department of Bioengineering, University of Washington, Seattle, WA, United States; ^3^Advanced Academic Programs, Krieger School of Arts and Sciences, Environmental Sciences and Policy, Johns Hopkins University, Washington, DC, United States

**Keywords:** non-ionizing electromagnetic fields, static/extremely-low frequency electromagnetic fields, radiofrequency radiation, wildlife, electro/magnetoreception, DNA, cryptochromes

## Abstract

There is enough evidence to indicate we may be damaging non-human species at ecosystem and biosphere levels across all taxa from rising background levels of anthropogenic non-ionizing electromagnetic fields (EMF) from 0 Hz to 300 GHz. The focus of this Perspective paper is on the unique physiology of non-human species, their extraordinary sensitivity to both natural and anthropogenic EMF, and the likelihood that artificial EMF in the static, extremely low frequency (ELF) and radiofrequency (RF) ranges of the non-ionizing electromagnetic spectrum are capable at very low intensities of adversely affecting both fauna and flora in all species studied. Any existing exposure standards are for humans only; wildlife is unprotected, including within the safety margins of existing guidelines, which are inappropriate for trans-species sensitivities and different non-human physiology. Mechanistic, genotoxic, and potential ecosystem effects are discussed.

## Introduction

Contrary to popular opinion, we know a great deal about how non-ionizing electromagnetic fields (EMF) affect non-human species because we have been using animal and plant models in research going back at least to the 1930's (1). Such research may have been conducted with humans in mind but can also be extrapolated to non-human species protection if we choose to apply it that way.

Mice and rats have been the primary animal species used in research, but also rabbits, dogs, cats, chickens, pigs, non-human primates, amphibians, insects, nematodes, various microbes, yeast cells, plants, and others. Effects have been seen in all taxa, in various frequencies, intensities, and exposure parameters. To non-human species, these are highly biologically active exposures, often functioning as stressors. This includes non-ionizing EMF in the static, extremely low frequency (ELF; 0–300 Hz) through the radiofrequency (RF) ranges used in all modern technology between 3 kHz and 300 GHz.

Extrapolations to wildlife from carefully controlled laboratory conditions, however, are difficult to quantify due to myriad variables such as: genetic variation and mobility, weather/climate change, site/region-specific environmental aspects, duration of exposure and variations in movements across habitats, species specialized physical characteristics, animal size, and orientation toward the field source—all of which can confound precise data assessment. Sometimes controlled studies correlate with patterns seen in wildlife, e.g., genetic, behavioral, reproductive, and other effects. Where this is the case, more confidence is possible. But often effects to wildlife manifest in the negative—species simply disappear. Nevertheless, increasing evidence has found effects to different species near communication structures in studies where extrapolations to field exposure have been made (2–9).

In addition, there have been extensive EMF wildlife reviews published between 2003 and 2021 (10–22). Recently, Levitt et al. (23–25) extrapolated to broad ecosystem level effects for the first time, including extensive tables that match rising ambient levels to effects seen at vanishingly low intensities now common in the environment as chronic exposures, and offer policy recommendations based on existing environmental laws.

The measured rising EMF levels in ambient environments (23) certainly elevate concerns, especially with 5G on the horizon using higher frequencies and novel signal characteristics/waveforms that are capable of affecting insects in particular with implications for the entire biome as discussed below. 5G is now increasing as a network platform in many places even as we are trying to figure out how to measure and distinguish its wideband signals from the larger scheme of 3–4G LTE networks with which it interacts. Already some of the unusual aspects of 5G (e.g., significantly higher peak emissions), are distinguishable from the background of other exposures as an environmental factor (26).

## Functioning misconceptions and terminology

There are two prevalent misconceptions today about how low-level non-ionzing EMF couples with and affects non-human species: (1) There is no need for environmental concern since exposures as currently regulated are too low to cause effects; and (2) Existing exposure standards for humans are sufficient to cover non-human species too. Neither supposition is accurate.

No radiofrequency (RFR) emission guidelines today take non-human species into consideration, despite constant measured rising background levels in urban, suburban, and rural areas [see Supplement 1 in reference (23)] that are capable of affecting wildlife and plants [see Supplement 3 and 4 in reference (24)]. This includes guideline allowances for RFR (100 kHz-300 GHz) created by the International Commission on Non-ionizing Radiation Protection (ICNIRP) (27), as well as a member organization of the American National Standards Institute (ANSI) called the International Electronics and Electrical Engineers (IEEE) that has written exposure guidelines for frequencies between 0 Hz and 300 GHz (28). Once countries or regulatory agencies, such as the U.S. Federal Communications Commission (FCC) (29), adopt such guidelines, they can become enforceable standards if those entities choose to do so within their statutory authority. The FCC can, and sometimes does, enforce RFR emission standards based partially on IEEE guidelines (For the purposes of this paper, we will refer to recommendations as exposure guidelines as applied to the environment). In addition, ICNIRP and IEEE/FCC only control for short-term acute exposures capable of heating tissue, not the long-term low-level chronic exposures common today for which they say there is not enough evidence to warrant change in recommendations (These authors disagree). They also fail to include important signaling characteristics (29), like modulation with significant biological effects particular to different transmission features (30). Many European countries, as well as Canada and Australia, have traditionally adopted ICNIRP guidelines (sometimes with slight variations) while others, like Switzerland, have adopted more stringent levels (25).

One complexity (among many) regarding writing EMF safety guidelines in general—but especially with wildlife in mind—involves the semantic difference between “emissions” (characteristics of the field at the transmission source) and “exposures” (the characteristics of the field absorbed by an object). ICNIRP/IEEE/FCC have guideline components for both emissions (expressed as a value of radiant energy in space for far-field encounters at some distance from the generating source) and exposures [expressed as a specific absorption rate (SAR) that is also pertinent to near-field exposures such as from cell phones held against the human head]. Emissions, of course, result in exposures; it is just a question of degree. Depending on species and environment, wildlife is capable of experiencing both near- and far-field exposures like humans. Once emissions leave the transmitting source, they are capable of creating broad exposures and becoming a chronic source of pollution. For the purposes of this paper, we will use “emissions” to denote transmission values and “exposures” to denote uncontrolled, unregulated ambient exposures.

There are many things in the environment that can affect how non-ionizing electromagnetic energy is absorbed, including atmospheric moisture and/or particulate content, soil composition, natural and/or artificial obstacles (trees/buildings), and the presence of other waveforms which can augment and/or diminish exposures, among others. Such complexities should not be used as an excuse to do nothing. Writing guidelines for all species is clearly a yeoman's task that will take far more than simply turning the power down; it may take significant electrical and RF re-engineering, alterations in frequency allocation, and societal change too (30).

## A current effort to include non-human species in emission guidelines

There is a current effort by the Australian Radiation Protection and Nuclear Safety Agency (ARPANSA), which uses the ICNIRP standards, to investigate broad information regarding effects to wildlife (31). ARPANSA, like ICNIRP/IEEE/FCC, has traditionally focused on human exposures with no recognized guidelines specifically addressed to the protection of plants and animals (31). The ARPANSA inquiry's emphasis thus far is on study design, i.e., how to sort research according to predefined inclusion/exclusion criteria, then incorporate the knowledge into “systematic maps” to see if the current human exposure criteria are sufficient to cover wildlife.

While this is a significant undertaking heretofore overlooked by guidelines-setting groups, the described approach may prove little more than a formula to verify the status quo. The defined exclusion criteria will likely eliminate from review most of the salient research on non-human sensitivity to the lowest intensity exposure levels to which many species are exquisitely sensitive at, or near, natural background levels that are clearly far below current guidelines. The resulting data will inevitably be skewed since the lowest level exposure research will be mixed in with controls and essentially disappear into the proposed analysis as a non-exposure, or it may be eliminated from review altogether. Example: the elimination criterion defines study controls as: “Sham exposure, no exposure beyond the background exposure level (which can be assumed to be negligibly low), or exposure at a lower level” (31). Since “lower level” is not defined and many ambient background levels are now artificially high [see Supplement 1 in reference (23)], this may not be the best methodology to quantify real-world field exposures to non-human species, let alone match it to relevant studies. Any true inquiry into EMF wildlife effects must begin from environmental/biological realities, not pre-existing dosimetry perspectives.

Different frequency ranges may adversely affect one species but have no impact on another. ICNIRP/IEEE/FCC's singular focus on heating effects may be particularly insupportable regarding insects, which can reach resonant matches with higher frequencies such as those used in the top ranges of 5G (>6 GHZ) due to insect's reduced size (32, 33). Insects do not dissipate heat and can suffer extreme effects within short periods of exposure even in much lower ranges (< 3 GHZ), leading to reproductive problems and death (1). Existing exposure standards may prevent humans from heating effects due to thermo-regulatory mechanisms but not with other species such as insects, small amphibians and reptiles.

Wildlife exposures today are just a question of degree. Many wildlife species constantly traverse varying artificial fields in all environments with many flying species—such as birds, bats, and insects—reaching extremely close proximity to transmission sources to which humans are rarely, if ever, exposed. Some of the highest power density areas, e.g., near broadcast antenna farms, are specifically located away from human populations with the assumption that if wildlife were impacted, they would abandon such sites for more favorable ones. But because of complex avian magnetoreception, RFR-generating infrastructure may be functioning as an attractant instead. Many such exposures may simply damage wildlife and go unnoticed, likely from near-field thermal effects as well as far-field non-thermal effects, among other causes (34–36).

Research on anthropogenic EMF has found non-linear effects that function differently from classic linear dose-response toxicology models. EMF effects may be fundamentally different than thermal effects, possibly working *via* different mechanisms (37). Effects may be more damaging to some species at lower intensities—the exact opposite of how emission guidelines that can become exposure standards are currently written and a primary reason to include the lowest level exposures in new research efforts. Even once pristine wilderness regions are now RFR-exposed environments from ground-based cell networks rimming national parks and wilderness areas, and from the exponential increase in satellites delivering internet connectivity to anywhere on Earth (23).

The true trans-species biological realities of today's exposures are enormously difficult to quantify, given the inherent variables of species differences, macro and microclimate adaptations, mating/migration patterns, and vastly different environments—e.g., aerial, ground-based, and aquatic—all with unique species-specific adaptions and electromagnetic receptor mechanisms. New methodological approaches that take the lowest exposures at ecosystem levels into consideration are needed.

## Natural sensitivities vs. manmade EMF

Many non-human species have highly specific vulnerabilities to anthropogenic EMF due to unique physiology that depend upon, and constantly use, the Earth's static geomagnetic fields for seasonal migration/orientation, nest/den building, mating, reproduction, offspring care, food finding, territorial defense, simple daily/seasonal circadian rhythms, and even longevity and survivorship. Electromagnetic perceptual factors include multi-system environmental species-specific mechanisms. Many species have specialized electroreceptor cells and/or magnetoreception abilities pertinent to their environments that far surpass human sensitivity. For instance, many species can sense natural DC magnetic fields in diverse ways including: migratory bird species (38, 39); numerous insect species including honey bees (40, 41); fish (42–47); mammals (48); bats (49); mollusks (50), and bacteria (51, 52). Some bird species may actually ‘see' the Earth's magnetic fields *via* complex magnetoception capabilities (53) located in their eye and beak areas.

As noted in Panagopoulos et al. (54), natural and manmade EMF are significantly and fundamentally different. Unlike natural EMF, all anthropogenic EMF is polarized, meaning it is more biologically active *via* the ability to amplify intensities (called constructive interference) as well as alter cellular charged/polar molecule oscillations into parallel planes in phase with the applied field. This can result in irregular gating in cell membrane ion channels and thereby disrupt the normal cellular electrochemical balance. In other words, manmade EMF can capture, entrain, and manipulate living cells' basic functioning architecture unlike natural EMF with which most living things have evolved. In addition, anthropogenic EMF typically functions at higher intensities for longer durations thereby increasing exposures in frequency ranges that are minimal in the natural environment, introducing signaling characteristics (modulation, phasing, pulsing etc.) that simply do not exist in nature but are now greatly amplified as a novel exposure due to technology. All these factors may account for the myriad biological effects seen in the literature over the last several decades.

## Magnetoreception: Mechanisms

There are three primary mechanisms involved with magnetoreception in non-human species:

- An induction process in which weak electrical signals are induced by magnetic stimulation in specialized sensory receptors (55).
- A magnetomechanical method in which localized deposits of single-domain magnetite crystals create signal information interactions (56, 57).
- A specialized-cell model in which radical-pair photoreceptor molecules create dedicated information pathways—an area getting significant research attention today (19, 30, 58–73).

In the induction model, according to Tenforde (57), specialized organs are involved with electrodynamic interactions with weak electromagnetic fields. In aquatic species this is seen in sharks, rays, and skates (elasmobranch fish) with heads that contain jelly-filled canals that have high electrical conductivity called Ampullae of Lorenzini. Small voltage gradients are induced in these canals *via* DC electric fields as low as 0.5 μV/m as these fish swim through the Earth's geomagnetic flux lines. Directional information is provided by the polarity of the induced field in relation to Earth's geomagnetic field. This may be an aqueous environment/species-specific factor as such organs have not been found in birds, insects, or land-based animals (58) although other physiological mechanisms may function in a similar capacity in some land-based species.

Many animals have evolved other special receptor organs. For example, the duck-billed platypus (*Ornithorhynchus anatinus*), a semi-aquatic egg-laying mammal, has thousands of electric sensors on its bill skin that allow for vital information processing in the somatosensory cortex (74). A platypus can detect an electric field of 20 μV/cm (equivalent to that produced by the muscles of a shrimp) *via* these electroreceptors interacting with a mechanoreceptor. Such electroreception is also seen in two aquatic species of monotremes: the long- bill (*Zaglossus bruijni*) and short-bill (*Tachyglossus aculeatus*) echidna. Other electric fish (including elasmobranchs) emit their own electric fields extending several centimeters for location/orientation, food-finding, and defense (75, 76). This unique ability allows electric fish to distinguish subtle differences in electrical properties within its immediate vicinity, including the electric fields of other fish, *via* electroreceptors capable of detecting a field of 5 nV/cm. While such evolutionary perceptual adaptations are extremely efficient and sensitive, they also render such species exceptionally vulnerable to unnatural anthropogenic fields. Some researchers postulate that electro-receptors in fish are a form of alternate touch and communication (77). The primary concern for aquatic species is from AC-ELF exposures from underwater cabling and other technologies, not RF which is of more concern for ground-based and aerial species (24).

The magnetomechanical model involves the naturally occurring iron-based crystal called magnetite (78–80) that has been found in most species studied, often in very different physiological areas. Magnetite-based orientation/interactions are patterned according to the geomagnetic field. Magnetite is highly reactive to external electromagnetic fields—a million times more strongly than any other known magnetic material. The abdominal areas of honey bees, for instance, contain magnetite with complex nerve endings feeding into it and can detect static magnetic field fluctuations as weak as 26 nT against background earth-strength magnetic fields that are much higher (79). They can also sense weak alternating fields at frequencies of 10 and 60 Hz (79). Bees are also affected by RFR as discussed below.

The third mechanistic model involves a complex conversion of electrons (singlet-triplet inter-conversion) and a free-radical-pair reaction in a group of proteins called cryptochromes.

As reviewed in Levitt et al. (24), cryptochromes have been found in the retinas of nocturnal migratory songbirds indicating intricate communication between avian eye and brain for orientation when relying on magnetoreception (38, 39). Cryptochromes were also found to be a critical magnetoreception component in fruit flies (*Drosophila melanogaster*) (81). Some other animals are also known to have retinal cryptochromes (38). Radiofrequency radiation (82) and oscillating magnetic fields have been reported to disrupt the migratory compass orientation in migratory birds (83). There are also reports of cryptochromes in plants which may account for the effect of EMF on plant growth (66). Cryptochromes are also known to be involved with circadian rhythms (72). Ritz et al. (63) published a review on the theories, plausibility, and complexities of cryptochrome/radical pairs.

Some species rely on combinations of mechanisms, e.g., two mechanisms exist side-by-side in some birds that mediate, as needed, different types of magnetic information. That is what facilitates flight on sunny vs. cloudy days and/or nocturnal flights. Both mechanisms can be easily disrupted (63, 84–86). It is thought that birds can co-process natural DC magnetic information with visual information and are able to distinguish them from each other (87, 88). According to Wiltschko and Wiltschko (88) and Wiltschko et al. (89), the likely mechanism occurs in the higher brain area and eyes *via* radical pair and light-dependent information processing (blue light absorbing photopigment cryptochromes have been found in avian retinas). The avian magnetic compass—an inclination compass—reacts to more than natural magnetic fields. RFR fields in the Larmor frequencies near 1.33 MHz were found to disrupt birds' orientation in an extremely sensitive resonance relationship (24). Radiofrequency radiation in particular may interfere with magnetoreception and be able to disable the avian compass while the exposures remain (4, 84). There are many uncertainties with this area in need of clarification.

Radiofrequency radiation may also affect natural “natal homing behavior”—the astounding ability of some species like sea turtles (90); eels (91); and salmon (42–44), among others—to return to their original birth location to reproduce. The underlying mechanism, though imperfectly understood, involves such species being “imprinted” with the exact location of their birth, likely through geomagnetic configurations, then “remembering” it at reproduction time even when thousands of kilometers away. The local geomagnetic field intensity and inclination angle are somehow impressed on newborns—information later used to return at breeding time. Landler et al. (92) found multiple effects of EMF in turtles that reproduce on land too, e.g., that RFR can alter natural orientation, establish its own orientation, and completely reverse natural orientation. This bellwether study is reason to protect sensitive breeding/nesting grounds from cell towers/transmitters being located nearby.

Different aspects of EMF and molecular mechanisms are likely used in many species and possibly more subtle stimuli as yet defined. The intensity and/or inclination of a stimulus, when combined with the vector of the geomagnetic field, may afford directional information. Avian behavioral studies (93) found birds used both cryptochrome and magnetite in response to a short intense pulsed magnetic field. It was also found that avian orientation was light-dependent and easily disrupted by high-frequency magnetic fields in the MHz range (83) suggesting that along with electrophysiological and histological studies, avian eyes have a radical pair mechanism providing compass-like directional information while magnetite in the upper beak senses magnetic intensity, thus providing positional information. The authors (83), however, pointed out that the songbird magnetic compass can be disrupted by an oscillating 1.403-MHz magnetic field of 2–3 nT—a level that cannot be explained by the radical-pair mechanism.

In 2014, Engles et al. (3) found magnetic noise between 2 kHz and 9 MHz disrupted the magnetic compass orientation of the migratory European Robin (*Erithacus rubecula*) at a vanishingly low level of 0.01 V/m, or 0.0000265 μW/cm^2^ (That frequency range is within AM radio transmission). Similar RFR magnetoreception interference has also been reported in the same species, with broadband being the most detrimental (8), as well as in other species (4).

Another long-distance migratory species—the iconic Monarch butterfly (*Danaus plexippus*) in the U.S.—is known to have magnetite in their antennae (94, 95) and to contain cryptochromes (96, 97). A 1982 study (98) found the head and thorax areas of monarchs contained magnetic materials and a 2014 study (99) found that monarchs' longest fall migration from Canada to wintering grounds in Mexico is assisted by a magnetic compass.

The above information indicates potential adverse effects at ecosystem levels to some avian, aquatic, and insect species from RFR at current ambient levels [see Supplement 1 in reference (23)].

## Genetic effects and EMF effects on insects

Despite classic assumptions that non-ionizing radiation cannot directly damage DNA, genotoxic effects have been seen in land-based, aerial, aquatic, and plant species at very low intensity RFR exposures far below ICNIRP/IEEE/FCC guidelines. There are at least 48 papers showing DNA damage after exposure to RFR at < 0.4 W/kg [see Supplement 1 in reference (24)]. Genotoxic effects are also seen in animal and plant species that are found exceptionally sensitive to both natural and man-made EMF [also see Supplement 2 in reference (24)]. Insects are of special concern as populations are being decimated globally (24).

At 1.2 MHz range—known as the Larmor frequency—insects demonstrated the strongest effects (100). The Larmor frequency is also related to radical pair resonance and superoxide formation. This indicates that RFR effects are frequency-dependent. 5G and broadband include this range. Extremely low frequency EMF was also found by Shepherd et al. (101) to disrupt the directional sense of honey bees *(Anthophila)*.

Depending on insect type and exposure duration, Michaelson and Lin (1) back in 1987 noted sequential insect reactions to RFR (at high intensities): insects first tried to escape, followed by motor disturbance and coordination problems, including stiffening, immobility, rigidity, and eventually death. At the same field intensity, *D. melanogaster*, for instance, survived longer than 30 min, whereas some tropical insects lived only a few seconds. Also seen were metabolic concentration changes and embryogenesis effects with gastrulation and larval growth being accelerated (102) (Embryogenesis is the period needed for a butterfly to complete metamorphosis). In 1961—in one of the earliest studies to find that pulsing alone is a biologically active exposure—Heller and Mickey (103) discovered that pulsed RFR between 30–60 MHz caused a 10-fold rise in sex-linked recessive mutations. In later studies using *D. melanogaster* models, Panagopoulos et al. (104) found severe effects in early and mid- stage oogenesis when flies were exposed *in vivo* to either GSM 900-MHz or DCS 1,800-MHz radiation from common digital cell phones, at non-thermal intensities for a few minutes per day during the first 6 days of adult life. The decrease in oviposition—as also previously reported by Panagopoulos et al. (105–107)—was hypothesized as due to degeneration of large numbers of egg chambers after DNA fragmentation of constituent cells. This was induced by both GSM and DCS mobile phone radiation. For the first time, induced cell death was documented in all cell types that constitute an egg chamber—including follicle cells, nurse cells, and the oocyte—and in all stages of early and mid-oogenesis from germarium to stage 10, during which programmed cell death does not physiologically occur (The most sensitive developmental stages to electromagnetic stress induced by the GSM and DCS fields were found to be germarium and stages 7–8). These papers, taken collectively, signify serious potential effects from cell phones/infrastructure and WiFi devices to all similar size insect species. Panagopoulos (108) further discussed the subject in an extensive review on genetic effects in 2019.

Ants also react adversely to RFR (109–111). Cammaerts et al. (111) found that memory and association between food sites and visual/olfactory cues in ants (*Myrmica sabuleti*) was significantly inhibited, with memory eventually wiped out altogether, from exposures to GSM-900 MHz signal at 0.0795 μW/cm^2^. A cumulative effect was seen even at very low intensity with subsequent exposure. The exposed colonies' overall condition eventually resembled that of honeybee (*Apis mellifera*) colony collapse disorder. The researchers concluded that exposures common to cell phones/towers and other transmission sources are capable of disastrous effects on a wide range of insects that rely on olfactory and/or visual memory, including bees.

For nearly 100 years, researchers have known that bees have an acute sense of the Earth's DC magnetic fields (40, 112–115) and rely on that perception for survival. Because of bees' outsize pollinator significance to human food supplies, and their current significant population declines, they are a much-studied model for ELF EMF and RFR effects (see below). Early studies were conducted in the ELF ranges (24) and are ongoing. For an excellent review of ELF/RFR-EMF effects to insects, including bees, see Balmori (16) and a recent article by Li et al. (114) for ELF-EMF exposure/developmental defects.

Some RFR effects seen in bees include: significant inhibitory effects on sensory olfactory excitability and short term memory impairment after 24-h WiFi-router exposure (116); induced worker piping—the sound that initiates swarming behavior in colonies, or as a warning/distress signal—that demonstrated 900-MHz GSM is a stressor to bees (117); reduced motor activity and changes in biomolecules in the body (118); reduction of worker bees and reduced egg laying by queens exposed to cell phone radiation (119); reduced hatching and altered pupal development after cell phone radiation exposure (120); decrease in comb weight and delayed return or hive abandonment after exposure to DECT phone radiation (121, 122); changes in carbohydrate, lipid, and protein concentrations in the body with cell phone radiation exposure (123, 124); and increased mortality with exposure to HF (13.56 MHz) and UHF (868 MHz) RFR (125). RFR has also been implicated in colony collapse disorder (117, 126, 127). Most of the above studies were conducted in non-thermal ranges and non-linear effects were often seen, with the lower exposures causing the greater effects.

Insect size, non-linear effects, waveform characteristics, frequencies, and RFR transmission direction/antenna tilt are critical concerns with 5G radiation today due to that technology's extremely complex near- and- far-field ambient exposures in all environments from ubiquitous macro- and micro-cells, as well as increased low Earth orbit satellite networks (23). The range of frequencies used for wireless telecommunication systems will increase up to 120 GHz for 5G from below 6 GHz for 3G, 4GLTE, and WiFi. The shorter wavelengths at such higher frequencies are a far better resonant match with small insects. Both heating and non-heating effects are likely to occur. Flora is also known to be adversely affected by RFR with implications for small cell placement on utility poles near trees [see Supplement 4 in reference (24)].

An alarming study by Thielens et al. (32) computer modeled (as a function of frequency alone) absorbed RFR from 2 GHz to 120 GHz in four different insect types. All insects indicated an increase in frequency-dependent absorbed RFR at and above 6 GHz compared to absorbed RFR below 6 GHz. Computer modeling demonstrated that an upward conversion to frequencies above 6 GHz at just 10% of the incident power density could lead to increased RFR absorption between 3-to-370%. This is a large differential indicating potentially serious consequences to numerous insect species and consequently the entire food web.

In 2020, Thielens et al. (33) investigated western honeybees (*A. mellifera*) with a combination of computer simulations and *in-situ* RFR exposure measurements near bee hives. Five models were exposed to frequencies already carved out for 5G—plane waves from 0.6 GHz to 120 GHz. Frequency simulations quantified averaged absorbed whole-body RFR. Depending on the specimen, they found the average increased by factors of 16-to-121 when a fixed incident electric field strength increased from 0.6 GHz to 6 GHz. Measurements were also taken near five different locations at 10 beehive sites. Results estimated a realistic absorption rate between 0.1 and 0.7 nW from an average total incident RFR field strength of 0.06 V/m, from which they concluded that an assumed 10% incident power density shift to frequencies higher than 3 GHz would cause increased RFR honeybee absorption between 390 and 570%. 5G involves just such a frequency shift.

The Thielens et al. (32, 33) studies alone raise serious concerns about ambient environmental invertebrate effects at these higher frequency exposures. There is a broad presumption of safety at ICNIRP/IEEE/FCC due to 5G millimeter-waves superficial penetration ability to affect skin tissue in humans. But shallow penetration in humans can equal whole body penetration in insects. This one technology has the ability to create significant holes in the food web with implications throughout the biome, yet no significant environmental reviews have been conducted prior to buildout and to date most emissions criteria adopted in various countries are primarily guidelines without consequence for violation.

## Discussion

It is clear that non-human species experience EMF as environmental stressors and biological effects can occur at anthropogenic levels in our present environment. This largely unrecognized variable can conceivably alter delicate ecosystems, arguably including the biosphere where all living organisms are located—and may, in fact, be doing so. Traditionally, other than in small localized situations, e.g., near powerline corridors or broadcast antennas, ELF/RFR-EMF environmental effects have not been of serious concern to regulating authorities. But this subject now requires immediate attention with 5G on the horizon, as well as a reexamination of chronic rising ambient levels across all non-ionizing electromagnetic frequency ranges today.

Investigators have known since the early 1970's how EMF and RF couples with most animal species (128, 129). Given our increasing ambient EMF levels, far more precise understanding of the molecular and cellular processes of electro- and magneto-reception in non-human species is suddenly critical. We may already be overwhelming some species' natural biological sensors that evolved over eons. Electroreception mechanisms, including magneto/electroreceptors, magnetite, and cryptochrome/radical-pairs, enable vast living organisms in all environments to detect the presence of, and immediate changes, in non-ionizing electromagnetic fields at very low intensities across a range of frequencies. Such heightened sensitivities function far beyond human perception and create unique vulnerabilities that can easily be disturbed by novel man-made fields. Since technology changes so fast, no evolutionary adaptation is possible.

Radiofrequency radiation is a form of energetic air pollution and should be regulated as such (25). U.S. law (130) [42 USC § 7602 (g)] defines air pollution as:

“The term “air pollutant” means any air pollution agent or combination of such agents, including any physical, chemical, biological, radioactive (including source material, special nuclear material, and byproduct material) substance or matter which is emitted into or otherwise enters the ambient air. Such term includes any precursors to the formation of any air pollutant, to the extent the Administrator has identified such precursor or precursors for the particular purpose for which the term “air pollutant” is used.”

Unlike classic chemical toxicology pollutants in which a culprit can typically be identified and quantified, RFR may function as a “process” pollutant in the air not unlike how endocrine dysruptors function in food and water in which the stressor causes a cascade of unpredictable systemic effects. The stimulus in the RFR analogy would be physical/energetic rather than chemical.

Long-term chronic low-level EMF exposure guidelines, which do not now exist, should be set accordingly for wildlife; mitigation techniques where possible should be developed; full environmental reviews should be conducted prior to the licensing/buildout of major new technologies like 5G; and environmental laws/regulations should be strictly enforced (25). We have a long over-due obligation to consider potential consequences to other species from our current unchecked technophoria—an obligation we have thus far not considered before species go extinct. In the views of these authors, the evidence requiring action is clear.

## Data availability statement

The original contributions presented in the study are included in the article, further inquiries can be directed to the corresponding author.

## Author contributions

All authors listed have made a substantial, direct, and intellectual contribution to the work and approved it for publication.

## Conflict of interest

The authors declare that the research was conducted in the absence of any commercial or financial relationships that could be construed as a potential conflict of interest.

## Publisher's note

All claims expressed in this article are solely those of the authors and do not necessarily represent those of their affiliated organizations, or those of the publisher, the editors and the reviewers. Any product that may be evaluated in this article, or claim that may be made by its manufacturer, is not guaranteed or endorsed by the publisher.
